# Hepatitis C Treatment Among Primary Care and Specialty Providers: A Single Center Study, 2015 to 2022

**DOI:** 10.1177/21501319241253521

**Published:** 2024-05-10

**Authors:** Anna Scialli, Sammy Saab, Anabel Salimian, Debika Bhattacharya, David Goodman-Meza

**Affiliations:** 1 University of California, Los Angeles, CA, USA; 2 Pfleger Liver Institute, Los Angeles, CA, USA

**Keywords:** hepatitis C, HCV, primary care, direct-acting antivirals

## Abstract

**Introduction:**

Despite national goals to eliminate Hepatitis C (HCV) and the advancement of curative, well-tolerated direct-acting antiviral (DAAs) regimens, rates of HCV treatment have declined nationally since 2015. Current HCV guidelines encourage treatment of HCV by primary care providers (PCPs). Payors have reduced restrictions to access DAAs nationally and in California however it remains unclear if the removal of these restrictions has impacted the proportion of PCPs prescribing DAAs at a health system level. Our objective was to examine the proportion of DAAs prescribed by PCPs and specialists and to describe the population receiving treatment in a single health system from 2015 to 2022.

**Methods:**

We examined the proportion of DAAs prescribed by PCPs and specialists and the population receiving treatment through a retrospective analysis of claims data in the University of California, Los Angeles (UCLA) Health System from 2015 to 2022. We described number of prescriptions for HCV medication prescribed by PCPs and specialists by year, medication type, and physician specialty. We also described numbers of prescriptions by patient demographics and comorbidities.

**Results:**

A total of 1515 adult patients received a prescription for HCV medication through the UCLA Health System between 2015 and 2022. The proportion of patients receiving prescriptions for PCPs peaked at 19% in 2016, yet decreased to 5.7% in 2022, an average of 13% across all years. Median age of patients receiving treatment was 60 years old, and 56% of patients receiving HCV treatment had commercial insurance as their primary payer.

**Conclusions:**

HCV treatment declined from 2015 to 2022 among specialists and PCPs in our health system. Older patients comprised the majority of patients receiving treatment, suggesting a need for novel approaches to reach patients under 40, an age group with significant increases in HCV transmission.

Hepatitis C (HCV) treatment has seen significant progress in the past decade. Direct-acting antivirals (DAAs) have demonstrated cure rates above 95% with simple and well-tolerated regimens.^1^ Despite these advances, approximately 2.2 million adults were living with HCV in the United States from January 2017 to March 2020.^2^ In California, there was a 15% increase in new diagnoses between 2014 and 2017.^3^ Meanwhile, the World Health Organization (WHO) and the Centers for Disease Control and Prevention (CDC) set a goal of HCV elimination by 2030.^4,5^ The National Academies of Sciences and Medicine (NASM) estimated that meeting this goal would require that 260 000 people receive HCV treatment in the US annually.^6^ Currently, there is significant political will to address HCV in the United States with President Biden’s announcement of a 5-year plan for $12.3 billion in funding aimed at reaching national elimination goals.^7^

A key strategy to increasing treatment for people living with HCV is for primary care physicians (PCPs) to initiate treatment. The American Association for the Study of Liver Disease (AASLD) and the Infections Diseases Society of America (IDSA) guidelines endorse the prescribing of DAAs by PCPs, citing the safety and efficacy of these treatments.^8,9^ Yet, while policies have encouraged the scaling up of treatment for HCV, treatment rates have steadily declined from 2015 to 2020.^10^ Treatment rates are less than one-third of the annual goal set by the NASM to achieve HCV elimination by 2030.^6,10^ Fewer than one-third of adults living with HCV receive treatment within a year of diagnosis, underscoring the persistence of barriers to care.^2^ Although research has found increases in the proportion of patients treated by PCPs, suboptimal access to care may persist due to PCP knowledge and administrative burden due to insurance claim denials.^11-13^ Ongoing reliance on specialists to provide HCV treatment presents challenges to patients, who may be lost to follow-up.^14,15^ Increasing access to DAAs through PCP prescribing could increase treatment, decreasing morbidity and transmission of HCV within the community.^16^

A previous study showed that the proportion of DAAs prescribed by PCPs increased since 2014 as policies emerged encouraging the treatment of HCV in primary care, however it is unclear if changes in rates of PCP prescribing are increasing at a health system level in the absence of targeted campaigns aimed at increasing HCV treatment rates.^11^ Further, little research exists examining patient characteristics among those who receive DAAs from PCPs versus those who are referred to specialists. We used the University of California Los Angeles (UCLA) Health system to examine how rates of HCV treatment and the proportion of PCP and specialist prescribing have changed since 2015 amidst a changing policy landscape surrounding DAA treatment.

## Methods

This was a retrospective study using data from UCLA Health electronic health records. Data was obtained from UCLA’s Office of Health Informatics & Analytics (OHIA) Data Repository & Dashboard (DDR). The DDR includes de-identified, system-wide patient encounters including demographic information, diagnosis codes, laboratory results, and prescription information for all UCLA health system hospitals and outpatient offices since 2013. This study was submitted to the UCLA Institutional Review Board (IRB) and was deemed exempt from IRB review due to data deidentification.

We included cases (1) aged 18 years or older, (2) that received HCV treatment, and (3) prescriptions were between 1 January 2015 and 1 January 2023. We excluded cases that received treatment as inpatients, or that received regimens containing ribavirin or pegylated interferon as these regimens would not be managed by PCPs.

We extracted demographic data (age, sex, and race/ethnicity), insurance status, comorbidities, and outpatient HCV medication prescribing data. Age was defined as the age in years at the time of prescribing an HCV medication. Race and ethnicity were recorded in the EHR and were categorized as Black, White, Asian, American Indian or Alaska Native, and Native Hawaiian or Other Pacific Islander. Ethnicity was recorded as Hispanic or Latino or non-Hispanic or Latino. Insurance status was coded by primary payer: dually insured Medicare and Medicaid patients were recoded as having Medicare insurance; dual private insurance and Medicare were recoded as privately insured. Comorbidities were diagnosed based on International Classification of Diseases-10 codes (ICD-10) and only comorbidities that were dated before the prescription of an HCV medication were included.

We defined HCV treatment as receiving a single prescription for a DAA medication for the treatment of HCV. These medications included ledipasvir-sofosbuvir, sofosbuvir-velpatasvir, glecaprevir-pibrentasvir, elbasvir-grazoprevir, sofosbuvir-velpatasvir-voxilaprevir, simeprevir-sofosbuvir, sofosbuvir-daclatasvir, and paritaprevir-ritonavir-ombitasvir plus dasabuvir. We assessed provider characteristics based on the encounter for the initial prescription of DAAs. We categorized providers as a specialist or PCP based on listed specialty as included in outpatient clinic visit data. We categorized providers with listed specialties of Internal Medicine, Family Medicine, or Medicine-Pediatrics as “primary care providers (PCPs).” We categorized all other providers as non-primary care providers or “specialists.”

We described HCV treatment by provider type and by year. We described patient characteristics using data included in clinical encounters including sex, age, language, race, ethnicity, and comorbidities. All analyses were conducted using R version 4.0.3.

## Results

From 2015 and 2022, 2315 cases received at least 1 prescription for an HCV medication. Of these, 800 cases were excluded as they were either under 18 years of age (5), initiated HCV medications as an inpatient (308), or received ribavirin (538) or pegylated interferon (5). Our final sample included 1515 cases.

**Figure 1.**
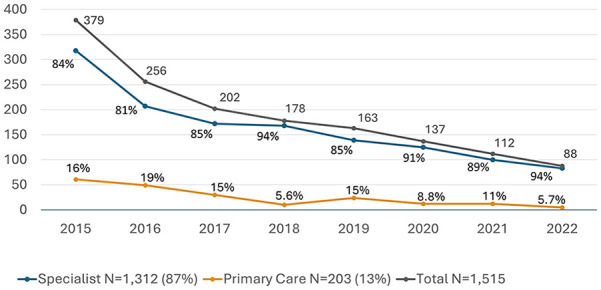
Specialist and primary care HCV prescriptions by year, 2015 to 2022. Pearson’s chi-square, *P* < .001. (Excel).

**Figure 2.**
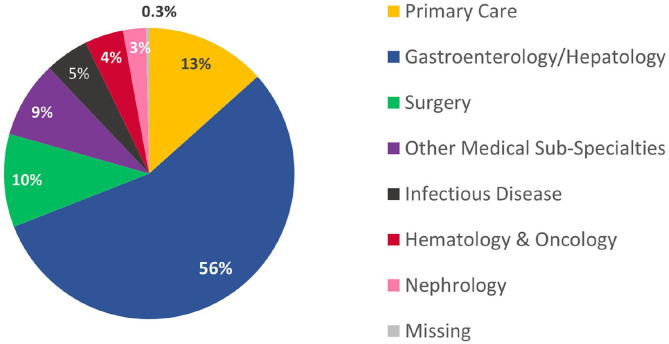
HCV prescriptions by provider specialty, 2015 to 2022. N = 1515. (Excel).

PCPs prescribed DAAs to 203 cases (13.4%) and specialists prescribed DAAs to 1312 cases (86.6%). There were 135 different PCPs that wrote prescriptions for HCV medication (median 1 case, range 1-9), and 289 different specialists that prescribed a DAA (median 1 case, range 1-245) during the study period. The number of patients receiving prescriptions for HCV treatment decreased in each year of the study period, declining from 379 in 2015 to 88 in 2022 (Figure 1). The proportion of patients receiving prescriptions from PCPs peaked at 19% in 2016 and decreased to 5.7% in 2022, with an average yearly proportion of approximately 12% (Figure 1). Of patients treated by specialists, which included any specialty aside from Internal Medicine, Family Medicine, or Medicine-Pediatrics, over half were treated by gastroenterologists (Figure 2).

Of patients who received HCV treatment from any provider type, 61% were male, 39% were female, and 0.1% identified as another sex or declined to answer (Table 1). Median age of patients receiving treatment was 60 years old (IQR: 52, 66) without significant difference in age of patients seen by specialists and PCPs. Differences in race were significant, however 30% of patients in our data did not have information on race, disproportionately comprising the patient group which were treated in primary care. The majority of patients receiving treatment from any provider had commercial insurance as the primary payer (56%, n=846.) No significant differences were found between patient insurance type and prescribing provider type.

**Table 1.** Patient Characteristics by Provider Type, 2015 to 2022.

	Overall N = 1515	Specialist N = 1312 (87%)	Primary care N = 203 (13%)	*P* value^a^
Sex				.08
Male	929 (61)	814 (62)	115 (57)	
Female	584 (39)	497 (38)	87 (43)	
Unknown	2 (0.1)	1 (<0.1)	1 (0.5)	
Age	60 (52, 66)	60 (52, 66)	60 (53, 67)	.86
Race				.02
American Indian, Alaska Native, Indigenous	11 (0.7)	11 (0.8)	0 (0)	
Asian, Asian American, Native Hawaiian, Pacific Islander	86 (5.7)	73 (5.6)	13 (6.4)	
Black, African, African American	137 (9.0)	110 (8.4)	27 (13)	
Multiple races	61 (4.0)	55 (4.2)	6 (3.0)	
Unknown, choose not to answer	449 (30)	406 (31)	43 (21)	
White, Caucasian, Middle Eastern, North African	771 (51)	657 (50)	114 (56)	
Ethnicity				<.001
Hispanic or Latino	279 (18)	254 (19)	25 (12)	
Not Hispanic or Latino	1006 (66)	843 (64)	163 (80)	
Unknown	230 (15)	215 (16)	15 (7.4)	
Language				.046
English	1390 (92)	1195 (91)	195 (96)	
Spanish	59 (3.9)	54 (4.1)	5 (2.5)	
Other	66 (4.4)	63 (4.8)	3 (1.5)	
Primary insurance				.65
Commercial	846 (56)	734 (56)	112 (55)	
Medicaid (Medi-Cal)	49 (3.2)	45 (3.4)	4 (2.0)	
Medicare	558 (37)	481 (37)	77 (38)	
Unknown	62 (4.1)	52 (4.0)	10 (4.9)	
Hepatitis B	73 (4.8)	66 (5.0)	7 (3.4)	.95
HIV	70 (4.6)	61 (4.6)	9 (4.4)	.89
Liver transplant	96 (6.3)	92 (7.0)	4 (2.0)	.006
Awaiting liver transplant	130 (8.6)	125 (9.5)	5 (2.5)	<.001
Cirrhosis	472 (31)	430 (33)	42 (21)	<.001
End-stage renal disease	109 (7.2)	103 (7.9)	6 (3.0)	.01
Dialysis	90 (5.9)	84 (6.4)	6 (3.0)	.05
Alcohol use disorder	137 (9.0)	118 (9.0)	19 (9.4)	.87
Opioid use disorder	87 (5.7)	71 (5.4)	16 (7.9)	.16
Stimulant use disorder	34 (2.2)	29 (2.2)	5 (2.5)	.80
Charlson-Quan Score	3.0 (2.0, 6.0)	3.0 (2.0, 6.0)	(2.0, 5.0)	.2

a Fisher’s exact test; Wilcoxon rank sum test; Pearson’s Chi-squared test.

Among patients receiving treatment from specialists, 33% had a diagnosis of cirrhosis compared to 21% of patients treated by a PCP (*P* < .001, Table 1). Similarly, 14% of patients in specialist care had hepatocellular carcinoma compared to 3.4% of patients being treated by PCPs (*P* < .001). Patients awaiting liver transplant and those with end-stage renal disease also comprised a bigger proportion of patients treated by specialists compared to primary care (Table 1). Of the entire study population, 8% (n = 121) had a diagnosis of opioid or stimulant use disorder. Please reference Table 1 for a full analysis of patient demographics and comorbidities by provider type.

**Figure 3.**
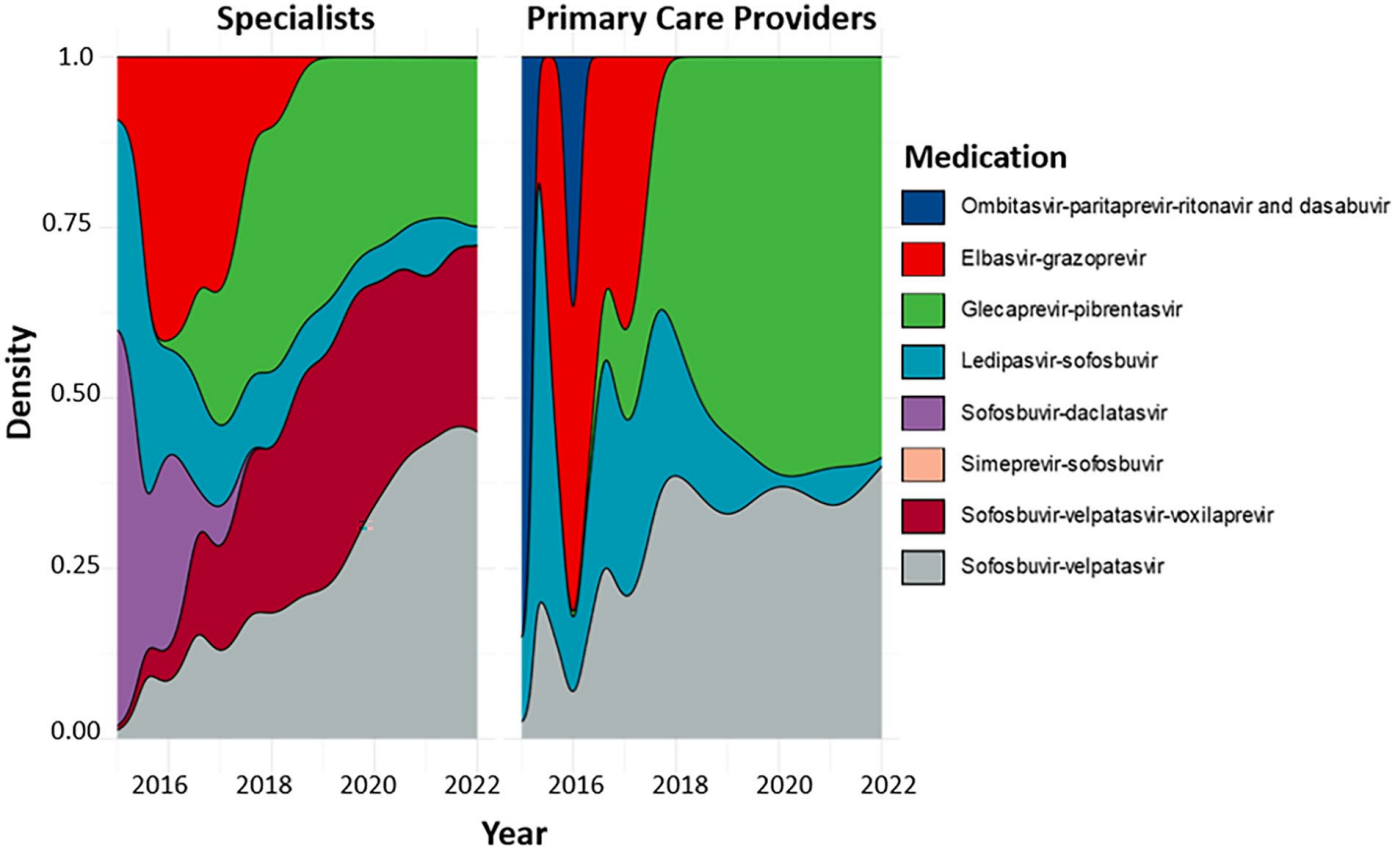
Density plots of medication prescribed by year by specialists and PCPs, 2015 to 2022. (R).

The majority of patients received prescriptions for ledipasvir-sofosbuvir (Harvoni, Gilead Sciences, Foster City, CA, USA), sofosbuvir-velpatasvir (Epclusa, Gilead Sciences, Foster City, CA, USA), or glecaprevir-pibrentasvir (Mavyret, AbbVie, North Chicago, IL, USA) regardless of provider specialty (Table 2). Over half of the patients treated by PCPs received prescriptions for ledipasvir-sofosbuvir (53.2%, n = 108, Table 2). Specialists prescribed 92% of sofosbuvir-velpatasvir prescriptions (*P* < .01) and 87% of glecaprevir-pibrentasvir prescriptions (*P* = .98). By the final year of the study period, glecaprevir-pibrentasvir and sofosbuvir-velpatasvir accounted for almost all prescriptions by PCPs (Figure 3).

**Table 2.** HCV Medication by Provider Type, 2015 to 2022.

	Specialist N = 1312 (87%)	Primary care N = 203 (13%)	*P*-value^a^
Ledipasvir-sofosbuvir	526 (83)	108 (17)	<.001
Sofosbuvir-velpatasvir	548 (92)	50 (8.4)	<.001
Glecaprevir-pibrentasvir	169 (87)	26 (13)	.98
Elbasvir-grazoprevir	30 (86)	5 (14)	.80
Sofosbuvir-velpatasvir-voxilaprevir	14 (93)	1 (6.7)	.71
Simeprevir-sofosbuvir	3 (100)	0 (0)	>.99
Sofosbuvir-daclatasvir	41 (82)	9 (18)	.33
Ombitasvir-paritaprevir-ritonavir and dasabuvir	7 (47)	8 (53)	<.001

a Fisher’s exact test.

## Discussion

During the study period, the proportion of new cases treated for HCV by PCPs decreased by a third. Changes in payor restrictions and requirements did little to shift new treatments to the primary care space in this medical system. While our study demonstrated greater primary care uptake of newer DAAs with simple treatment regimens, this did not translate into a higher proportion of PCPs treating patients with HCV in this health system. Further research is needed to evaluate the root causes and to identify potential interventions to increase HCV treatment in the primary care setting, whether through increasing treatment of patients already diagnosed with HCV or increasing identification of patients living with HCV without a diagnosis. Prior research found that 71% of surveyed PCPs refer patients with HCV to specialty care, primarily due to lack of familiarity with treatment regimens, lack of time, and competing priorities.^17^ Models such as the Extensions for Community Health Outcomes (ECHO) Project, in which PCPs attend trainings and consult with specialists via telehealth, have increased odds of receiving treatment among patients in primary care.^18^ Other models include an eConsult service, in which PCPs receive individualized treatment recommendations from specialist pharmacists and providers. Piloted in the San Francisco Health network, this model tripled the number of patients treated in primary care.^19^

Provider training may not fully address barriers to PCPs providing HCV treatment. A Virginia study on a program to increase provider efficacy through an in-person HCV training program found that a minority of providers began prescribing HCV independently after training, suggesting there may be other provider or system level barriers to providing HCV treatment in primary care.^20^ Prior research has shown that providers cited concerns about adherence among patients with a substance use disorder (SUD) as a barrier to providing treatment,^21^ despite evidence showing high treatment completion rates among these patients.^22^ Ultimately, attitudes of health system providers toward HCV treatment remain unknown, and more research is needed to fully understand the barriers to providing HCV in this particular health system.

Injection-drug use is the most commonly reported risk factor among new cases of chronic HCV,^23^ with an estimated 23% of new HCV cases occurring due to transmission through injection equipment.^24^ Fewer than 10% of patients in our study had a diagnosis of opioid or stimulant use disorder, suggesting that individuals who use drugs are not encountering the health system, are not being identified by physicians, or are not receiving treatment. This gap may be due to socioeconomic barriers to receiving healthcare but could also be explained by patient avoidance of health care and hesitance to disclose injection drug use due to stigma and provider discrimination experienced by people who use drugs.^25^ Emerging research has explored the efficacy of co-locating HCV treatment in addiction medicine clinics as well as integrating both SUD treatment and HCV treatment in primary care settings, offering potential avenues in which health systems could identify patients at high-risk for HCV and provide treatment.^26^ Interventions aimed at identifying those with HCV who may not otherwise interact with the healthcare system include mobile and street-based clinics, such as the UCLA Health mobile clinic that provides testing and treatment for HCV.^27^

Overall, HCV treatment numbers decreased over time regardless of provider type. Despite increased rates of incident HCV cases in California from 2014 to 2017,^3^ there were decreasing numbers of new patients treated for HCV at our health system since 2015 in both specialty and PCPs. This decrease may be due to the changing HCV treatment landscape early in our study period. During this time there was an expansion in more effective and less toxic medications,^28^ loosening of treatment restrictions in California Medicaid (Medi-Cal),^29^ as well as increased reimbursements for HCV screening and treatment authorization for Medicare members.^30,31^ Large numbers of patients receiving treatment at the beginning of the study may be attributable to a “backlog” effect seen in states that had substantial HCV policy changes, in which patients who had known HCV diagnoses were quickly connected to treatment once treatment eligibility restrictions were relaxed.^32^ After this initial increase in treatment, rates declined. This may also account for the age discrepancy in our study, as from 2012 to 2020, CDC screening guidelines only indicated universal HCV screening for individuals born from 1945 to 1965.^33^ Patients within the “baby boomer” cohort were therefore more likely to be screened prior to and during much of the study period. These patients may also be more likely to be receiving care from specialists due to comorbid conditions and thus be driving HCV treatment into specialty care, however more analysis into this area is warranted. This overall decreasing trend in treatment is consistent with research from Teshale et al,^11^ which observed decreases in national treatment rates since 2015 despite new screening guidelines and reduced treatment restrictions.

Decreasing rates of treatment may also be attributable to the fact that few patients in our study were covered by Medicaid insurance. Nationally, Medicaid covered 79% of people who tested positive for HCV from 2019 to 2022,^2^ suggesting many Californians with HCV would not be screened, identified, or treated by health system providers. In 2021, new HCV Medicaid infections were highest among individuals aged 30 to 39 years (3.5/100 000) and American Indian/Alaska Native individuals (2.7/100 000), groups that were underrepresented in our study data.^23^

There were several limitations of our study. This was a single center study restricted to Los Angeles, California, which limits the generalizability of the findings to other health systems and geographic regions. Data was unavailable for patients that may have received treatment at other medical centers. Our data was unable to show when patients were referred to non-UCLA providers for HCV treatment, thus missing encounters in which patients successfully were linked to treatment outside of the UCLA system. Race and ethnicity were inconsistently collected, with 30% and 15% of data missing respectively. It is also unclear what training for HCV treatment has been offered to PCPs in the UCLA Health System during the study period. Finally, we did not have data about HCV risk factors, particularly injection drug use. Research has demonstrated that ICD codes are insufficient to identify injection drug use, highlighting the need for improved data to better understand clinical decision making for patients who are identified as persons who inject drugs.^34^

## Conclusion

In summary, we found that since 2015, the rate of HCV treatment has not increased in primary care settings within the health system studied. This data adds to existing evidence that the “baby boomer” cohort and those with private or Medicare insurance comprise the majority of those that received treatment shortly after the introduction of DAAs.^2,10^ This finding is in contrast to the demographic currently most impacted by HCV: individuals aged 21 to 30 who inject drugs.^3,24^ System-wide efforts to reach, screen, and treat individuals in this group are necessary to meet national elimination goals. Interventions aimed at increasing provider efficacy and reducing provider bias may also be instrumental in increasing PCP’s motivation to identify and treat patients with HCV. However, alternative strategies outside of traditional clinical settings may be necessary to achieve HCV elimination goals. Offering care in settings that reduce barriers to treatment, such as mobile-based clinics, is a promising strategy to engage with patients who face barriers to entering or completing the HCV care cascade. As funding and political will to eliminate HCV increase, more information is needed to understand how to facilitate identification and treatment of people with HCV by the UCLA Health System, especially among groups most impacted by HCV.
